# Fungal Pathogens and Seed Storage in the Dry State

**DOI:** 10.3390/plants11223167

**Published:** 2022-11-18

**Authors:** Isaura Martín, Laura Gálvez, Luis Guasch, Daniel Palmero

**Affiliations:** 1 Plant Genetic Resource Centre (CRF), National Institute for Agricultural and Food Research and Technology (INIA-CSIC), 28805 Alcalá de Henares, Spain; 2 Department of Agricultural Production, School of Agricultural, Food and Biosystems Engineering, Universidad Politécnica de Madrid, Avda. Puerta de Hierro, 4, 28040 Madrid, Spain

**Keywords:** seed-borne fungi, seed storage, gene bank, seed health methods

## Abstract

Seeds can harbor a wide range of microorganisms, especially fungi, which can cause different sanitary problems. Seed quality and seed longevity may be drastically reduced by fungi that invade seeds before or after harvest. Seed movement can be a pathway for the spread of diseases into new areas. Some seed-associated fungi can also produce mycotoxins that may cause serious negative effects on humans, animals and the seeds themselves. Seed storage is the most efficient and widely used method for conserving plant genetic resources. The seed storage conditions used in gene banks, low temperature and low seed moisture content, increase seed longevity and are usually favorable for the survival of seed-borne mycoflora. Early detection and identification of seed fungi are essential activities to conserve high-quality seeds and to prevent pathogen dissemination. This article provides an overview of the characteristics and detection methods of seed-borne fungi, with a special focus on their potential effects on gene bank seed conservation. The review includes the following aspects: types of seed-borne fungi, paths of infection and transmission, seed health methods, fungi longevity, risk of pathogen dissemination, the effect of fungi on seed longevity and procedures to reduce the harmful effects of fungi in gene banks.

## 1. Introduction

Seeds have been the basis for agriculture and foodstuffs since Neolithic times. Most important crops are grown from seeds, and cereal and legume grains constitute the main raw material of human food and animal feed. Seeds can harbor a wide range of microorganisms, especially fungi, which can cause significant sanitary problems in the seeds themselves, in growing crops, as well as to human and animal health. The main harmful effects of seed-associated fungi are mainly related to the transmission and dissemination of plant diseases, the reduction of seed quality and seed longevity, and the production of mycotoxins that can be highly toxic to humans and animals through the consumption of contaminated materials.

Seeds can be passive carriers of pathogens which can be transmitted to growing plants when environmental conditions are suitable. The term seed-borne describes the state of any microorganism that is carried with, on, or in the seed. The term seed-transmitted refers to the act of infecting seedlings from seed-borne inoculums. The first records of seed-borne diseases date back to the middle of the 18th century and reported bunt and smut diseases of cereals. One century later, the fungus causing bean anthracnose was demonstrated to be seed-borne [1]. At the beginning of the 20th century, the growth of the seed industry and the global seed market worldwide were the most likely causes of many new outbreaks of diseases [2]. In the following years numerous examples of destructive diseases disseminated by seeds were reported [3]. Although to a lesser extent, the increase in the international exchange of plant germplasm for breeding programs and research purposes in the last few decades has also increased the risk of spreading seed-borne pathogens into new areas [4,5].

Many seed-borne fungi may drastically reduce the germination of seeds stored for genetic preservation or plant reproduction [6]. It has long been known that some of the fungi that infect seeds before harvest, in the field, can reduce seed quality. However, the effects of other fungi that mainly invade seeds after harvest, the so-called “storage fungi”, were not reported until the 1940s [7].

Mycotoxins are secondary metabolites produced by filamentous fungi capable of causing serious diseases in humans and animals [8]. The major mycotoxin-producing fungi are species of *Aspergillus*, *Fusarium*, *Alternaria* and *Penicillium*. The mycotoxins that pose the greatest potential risk as food and feed contaminants are aflatoxins, altersolanol, trichothecenes, fumonisins, zearalenone, ochratoxin A and ergot alkaloids, among others [9]. Mycotoxigenic fungi are generally not aggressive pathogens, but they are often well-adapted to growing on substrates with low moisture, and they can colonize stored seeds. A major concern related to mycotoxins arose from the discovery of aflatoxins in poultry feed in England in the early 1960s [10], and since then extensive research has been carried out in this area [11].

In this review, an outlook on some basic aspects of seed-borne fungi, such as the fungal types and pathways of infection and transmission, has been included to help the understanding of the interactions and processes that occur between fungi and seeds. An overview of seed health methods is also provided, from conventional techniques to the latest molecular analyses, as early detection is a key aspect for controlling problems caused by seed-borne pathogens.

This review focuses especially on gene bank seed storage, as the role of fungi in seed germplasm collections has been much less studied than in commercial seed storage and presents particular aspects that should be taken into account. The conditions used in gene banks to prolong the lifespan of the seeds may also favor the survival of seed mycoflora. Thus, germplasm collections can act as pathogen reservoirs, and, therefore, gene banks should adopt measures to minimize the negative effects of fungi on both preserved and distributed seeds. To address this issue, general guidelines are given for the different stages of gene bank seed conservation.

## 2. Seed-Borne Fungi

A vast number of seed pathogens are described in the scientific literature. Neergaard [3] included in his reference book a survey of seed-borne fungi, and more than a thousand microorganisms, most of them fungal, are listed in the ISTA publication “An annotated List of Seed-borne Diseases” [12]. An update of this list is available in the “ISTA Reference Pest List” (https://www.seedtest.org/en/pest-list-tool-_content---1--3477.html, accessed on 1 September 2022). For vegetable species, ISF (International Seed Federation) has set up a regulated pest list database (https://pestlist.worldseed.org/public/pestlist.jsp, accessed on 1 September 2022). Other practical on-line information on relevant seed pathogens of the world’s major crops can be found in the “Safe Transfer of Germplasm Guidelines” section of the CGIAR (Consultative Group on International Agricultural Research) “Crop Genebank Knowledge Base” (http://cropgenebank.sgrp.cgiar.org, accessed on 1 September 2022). Information about seed-borne pathogens of forest tree seeds is given in a specific IPGRI Technical Bulletin [13].

### 2.1. Types of Seed-Borne Fungi

From an academic point of view, taxonomic criteria would be the primary way to classify seed-borne fungi. Fungal taxonomy and nomenclature are complicated disciplines that have changed remarkably over the past decades, mainly due to advances in molecular biology techniques [14]. Microorganisms traditionally known as fungi are now spread across three kingdoms: Fungi (true fungi), Chromista, and Protozoa [15] and the organisms belonging to the two last kingdoms are commonly called fungal-like organisms or pseudofungi. Most seed-borne pathogens are included within the Fungi kingdom but some oomycetes (Chromista kingdom), such as *Phytophotora*, *Phytium* or *Peronospora*, are also important disease causative agents.

The nomenclature of fungi is also especially complex due to existing synonymies and the dual system that employed different binomial names for the asexual (anamorph) and sexual (teleomorph) forms of the same species. Valid names and taxonomic classification of fungi can be accessed in the database “Species Fungorum” at (http://www.speciesfungorum.org, accessed on 1 September 2022). In this work, fungi are generally designated by the most known name or by the name given by the cited author, although in some cases alternative names are included in brackets to facilitate identification.

From an economic point of view, seed-borne fungi can be classified under three groups: (a) plant pathogens that are transmitted by seeds, (b) fungi that reduce seed and grain quality, and (c) fungi that have no detrimental effects.

(a) Seed-transmitted pathogens can be categorized into two broad groups—biotrophs and necrotrophs—[1]. Biotrophs are obligate parasitic organisms that develop specialized infection structures (haustoria) and live on nutrients provided by the living host. They have a narrow host range and usually cannot be grown in pure culture. Necrotrophic organisms destroy the host cells and then live saprophytically on the dead tissues. They kill their hosts by secreting toxins or cell-wall degrading enzymes and eliciting reactive oxygen species (ROS) production [16]. Necrotrophs frequently have a wide host range and can be grown in pure culture.

Although some fungi, such as powdery mildews, downy mildews (Oomycetes) or smut diseases, are obligate biotrophs [17], most seed-borne fungi are necrotrophs. Biotrophy and necrotrophy should probably be viewed as a continuum [18], and between these two groups there are also intermediate parasites (hemibiotrophs) which start off as biotrophs and eventually become necrotrophic, employing tactics from both pathogen groups. *Colletotrichum* spp. [19], *Fusarium* spp. [20], *Cochliobolus* [21] or *Septoria* spp. [22] are examples of hemibiotrophic fungi.

(b) Seed fungi that reduce seed and grain quality are commonly divided into two ecological general groups—field fungi and storage fungi—depending on whether the infection occurs mostly before or after harvest. Field fungi invade seeds before harvest when seeds are developing on mother plants in the field or after they have matured. In order to grow, these fungi require a seed moisture content in equilibrium with a relative humidity of at least 90–95%. This would correspond to a “water activity” (a_w_) equivalent to 0.90–0.95. Water activity is a criterion often preferred to seed moisture content and corresponds to the relative humidity of the air in equilibrium with the seeds (divided by 100) [6]. *Alternaria*, *Fusarium*, *Cladosporium* or *Helminthosporium* (*Bipolaris*/*Deschlera*) are very common field fungi [7]. Damage caused by field fungi usually occurs before harvest and does not increase in storage. Field fungi may discolor seeds, cause the weakening or the death of embryos and generate compounds toxic to man and animals. Some of them can also be transmitted and cause diseases on new seedlings or growing plants.

The severity of field fungi infections depends greatly on the environmental conditions during seed development. In general, the incidence of these diseases increases when prolonged wet periods occur during seed maturation phases [23,24,25]. Differences in weather conditions from one year to another, planting and harvest dates, planting densities or insect damage may influence the degree of infection [26,27,28]. There can also be considerable variations among cultivars in their susceptibility to seed infection [29,30,31].

Storage fungi invade seeds during storage and are not usually present to any great extent before harvest. The most common storage fungi belong to the genera are *Aspergillus* and *Penicillium* and they grow at seed moisture contents in equilibrium with relative humidities from 65 to 90% (Table 1). Other less relevant species that can invade stored grains are *Rhizopus*, *Mucor,* or *Chaetomium*. Storage fungi are saprophytes or weak pathogens widely distributed and almost always present. The major effects of these fungi are the discoloration of embryos, decreases in germinability and the production of mycotoxins. When the moisture content is high, they can also cause spontaneous heating as a result of a significant increase in respiratory activity, which is usually accompanied by a drastic reduction in seed quality [6,7].

The distinction between field and storage fungi is not always clear. For instance, the common storage fungus *Aspergillus flavus* can colonize maize kernels and groundnut seeds in the field and lead to serious problems of aflatoxin contamination [33,34,35]. Certain *Penicillium* species are also found as field fungi, and *Fusarium* spp. can continue to decay grains in storage if the moisture content is high enough [32].

(c) A large number of seed-borne fungi have never been shown to cause damage as a result of their presence in seeds [36]. In fact, many of them have a positive relationship with plants, such as some endophytes of grasses [37,38]. Others compete with pathogenic microorganisms [39,40,41]. Finally, some fungi, such as *Trichoderma* spp., are even used as seed treatments to control diseases enhancing plant growth [42]. Although beyond the scope of this review, seed microbiome is an emerging field of study that should be investigated in depth [43].

### 2.2. Paths of Infection, Inoculum Location and Disease Transmission

Pathogenic fungi have different mechanisms to gain access to the seed and can be located in different parts of the seed. Some of them are able to affect seedlings and cause damping off or develop diseases in the next plant generation. A brief summary of these aspects is given below, and detailed descriptions can be found in the classical books of Neergaard [3], Maude [1], and Agarwal and Sinclair [44].

In the field, fungal infections can reach the ovule or seed at any stage from the initiation of the ovule to the mature seed. The infection can be directly transmitted to the seed from the mother plant in a systemic way, or it may come from external sources, carried by the wind, rain, or insects [45]. Seed–transmitted vascular wilts (special forms of *Fusarium oxysporum*, *Verticilium* spp.) and the endophytes of some grasses are examples of fungi that produce a systemic infection in seeds via the vascular system or the tissues of the mother plant [46,47]. An indirect systemic infection occurs when the inoculum enters through the stigma and follows the same path as the pollen grain. Infection via the stigma can take place in the loose smuts of wheat and barley (*Ustilago tritici* and *U. nuda*) or in the internal mold of peppers (*Alternaria alternata*) [48]. Finally, many fungi invade the ovary/fruit walls from infected parts of the flowers or from spores disseminated by air and other agents, and then enter the seed through natural openings or breakages. Many necrotrophic fungi (e.g., *Botrytis*, *Alternaria*, *Diaporthe*, *Cercospora*, *Ascochyta* and *Phoma*) follow this infection route. The same pathogen can infect the seed using several mechanisms.

After seed maturation, there are further possibilities of infection or contamination during harvest, post-harvest operations and during seed storage. For instance, plant residues, soil or processing equipment may be agents of seed contamination. Seeds can become infected during storage if seed moisture contents are not low enough to inhibit the growth of *Aspergillus* spp. and other xerophytic fungi (Table 1). Contamination occurs through small quantities of spores that may be present in seeds before harvest or from spores already in storage facilities. This small amount of inoculum can increase rapidly under high temperature and moisture conditions.

Seed-borne fungi can be carried by seeds externally or internally, as spores or resting mycelia. A seed is said to be infested or contaminated when the fungi is adhered to the seed surface, mixed with seeds as sclerotia or found in soil particles or plant debris. The seed is infected when the pathogen has penetrated the seed tissues.

Biotrophic organisms, including most seed-borne viruses, many bacteria and some specialized fungi are usually located in the embryo. Classical examples of embryo-borne fungi are the loose smuts of wheat and barley [49]. Necrotrophic fungi that infect seeds are generally located in the seed coat or pericarp tissues. Deeper penetration is infrequent, although severe infections can result in the invasion of all seed parts including the embryo. Storage fungi may infect seed coats and embryos, with differences in susceptibility among plant species [50]. The embryos of cereal grains are readily invaded by storage fungi [51].

Seed transmission of disease occurs when the seed-borne inoculum is transferred to growing seedlings. This process may be systemic, when pathogens invade the seedlings and develop within them, or non–systemic, when microorganisms spread to seedlings and cause local infections. Both types of infections can start from fungal spores or mycelia inside the seeds or on the seed surface. Examples of seed fungi that cause systemic infections are the common bunt of wheat (*Tilletia caries* and *T. laevis*) and the loose smut of wheat and barley. Non-systemic infections are transmitted by contact and follow a discontinuous pattern. Seedlings may be killed before they emerge or blighted. Many fungal species of *Alternaria*, *Bipolaris*, *Cladosporium*, *Fusarium*, *Botrytis*, *Cercospora*, *Phoma*, *Ascochyta*, *Colletotrichum,* and *Septoria* are examples of fungi that cause non-systemic infections.

## 3. Seed Health Methods: An Outline

The detection and proper identification of causal organisms are essential tasks for the control of seed-borne diseases. Seed health was an early concern of the International Seed Testing Association (ISTA), and the Seed Disease Committee (at present Seed Health Committee) was established at the end of the 1920s with the main purpose of standardizing and improving diagnostic methods for seed pathogens [52]. The “Manual for determination of seed-borne diseases” published in 1938 by L.C. Doyer [53], the first chairman of the Seed Disease Committee, was the first publication to describe reproducible testing procedures for detecting fungi in seeds. Since then, several other important publications have described classic diagnostic methods [1,3,44,54]. ISTA-validated methods [55] are available on the website: https://www.seedtest.org/en/international-rules-for-seed-testing/seed-health-methods-product-1054.html (accessed on 1 September 2022). At the time of writing this chapter, there are 34 reference protocols listed on the ISTA website, among those, 22 are methods to detect fungi in seeds. Other organizations that publish standardized seed health test methods are ISHI-Veg (International Seed Health Initiative for Vegetable Crops) and USDA’s NSHS (National Seed Health System). Specificity, sensitivity, speed, simplicity, cost effectiveness, and reliability are mean requirements for the selection of seed health tests methods [56].

### 3.1. Conventional Methods

**Figure 1:**
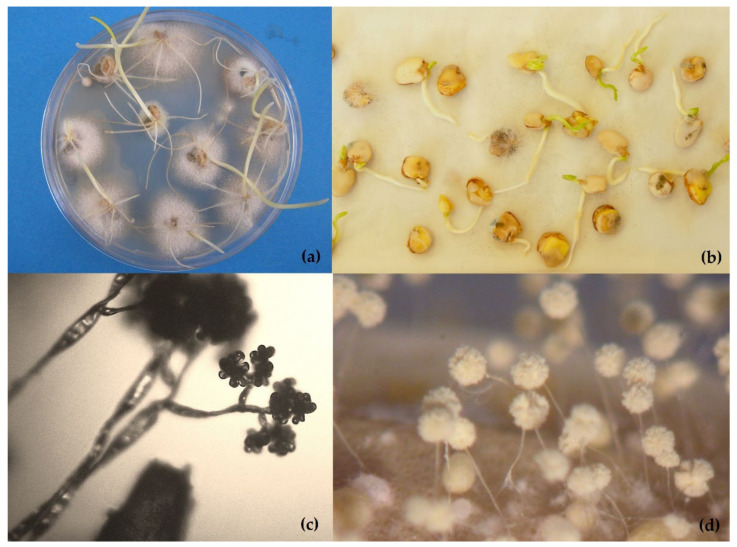
Conventional methods of detection of fungal pathogens in seeds: (**a**) wheat seeds infected with *Fusarium* under agar media test; (**b**) germinating seeds of *Lathyrus sativus* under blotter testing method after 40 years of storage at the CRF-INIA gene bank; (**c**) morphology of conidiophores and typical conidia of *Botrytis* spp.; (**d**) *Aspergillus* spp. by optical microscope.

Traditional methods include the direct inspection of dry seeds to observe fungal structures and seed symptoms, washing tests to detect spores located on the seed surface, incubation methods in agar media or on filter paper (blotter tests), staining methods or embryo extraction for seed-borne biotrophs which cannot be grown on external substrates, seedling symptom tests, and pathogenicity tests with plant inoculation. These methods are still the basis of seed health testing, but they are both time- and space-consuming, require specialized taxonomical expertise and are sometimes not sensitive enough to detect low levels of infection. All validated methods included on the ISTA protocols are direct methods based mainly on the agar plating (Figure 1a) or blotter assay (Figure 1b). Different culture media such as PDA (Potato Dextrose Agar) or MA (Malt Agar) are also used. Media are sometimes supplemented with streptomycin sulphate to limit bacterial growth. Sometimes it is necessary to carry out previous disinfection treatments for seeds with sodium hypochlorite solution (1% available chlorine), hydrogen peroxide (30%), or heat treatment to reduce the growth of saprophytes. Moreover, the abundant development of saprophytic molds including “storage fungi” in tests can be an indication that the seed is not of good quality due to unfavorable harvesting, processing, or storage conditions, or due to ageing. Some fungi (such as *Rhizopus* spp.) spread rapidly over tests on blotters and may interfere with outgrowth of the pathogen from the plate infected seeds [55]. Fungal identification is made by direct examination of the fungi colonies and the morphology of microsclerotia, conidiophores and conidia on culture media with an optical microscope (Figure 1c,d).

### 3.2. Serological and Molecular Methods

In the 1980s, new diagnostic technologies based on serological characters and DNA analyses were developed. Serological seed assays rely on antibodies generated against unique antigens of plant pathogens and include the enzyme-linked immunosorbent assay (ELISA). Serology is the most widely used detection assay for seed-borne viruses and has been employed to detect seed fungi pathogens [57,58], although the unavailability of species–species antibodies is a limitation [59]. In areas with limited access to facilities for molecular methods, ELISA methods offer laboratories affordable possibilities of sensitive pathogen detection [60].

**Figure 2:**
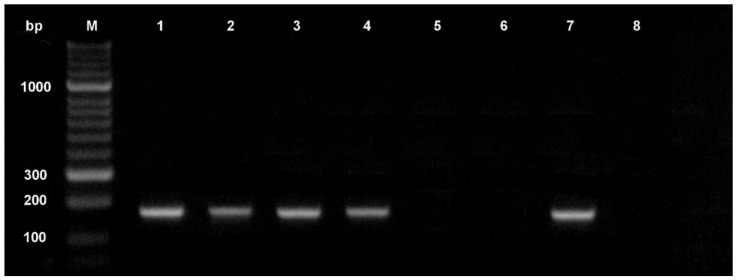
Detection of *Fusarium oxysporum forma specialis lactucae* race 1-specific DNA fragment in lettuce seeds using PCR assay. Lanes: M, HyperLadder 50 bp DNA ladder (Bioline), 1–4 *F. oxysporum* f. sp. *lactucae* race 1 isolates; 5–6 non-pathogenic *F. oxysporum*; 7 positive control (*F. oxysporum* f. sp. *lactucae* race 1 DNA); 8 negative control (water replaced genomic DNA as the template).

Molecular methods based on the polymerase chain reaction (PCR) have potential advantages over conventional detection methods, since they are more sensitive, specific, and faster, and they can be used by personnel with little experience in pathogen identification based on morphological characters [61]. Techniques based on the PCR have been used for many years for different applications in seed health testing, including identification of pathogenic fungi (Figure 2). These assays allow identification of non-infected seed lots more rapidly than direct tests (agar plating or blotter assay). Both ELISA and PCR techniques provide an indirect measure of signaling the presence of the pathogen by identifying the presence of pathogen-specific proteins or nucleic acids, but these indirect tests used for pathogen detection do not provide any information about pathogen viability. According to the International Seed Health Initiative for Vegetable Crops (ISHI-Veg), a negative result in an indirect test requires no additional testing to confirm that the seed lot in question is healthy. Instead, a positive result only indicates that the seed lot is suspected of being infected with the target pathogen, and a confirmatory test that shows the target pathogen to be viable and pathogenic must be followed [62].

The development of molecular detection procedures is a continuous race to optimize sensitivity and specificity. In the last few years, PCR assays have been improved with upgraded techniques, such as nested PCR, multiplex PCR, real-time PCR, magnetic capture hybridization (MCH)-PCR and biological and enzymatic polymerase chain reaction (Bio-PCR) for detecting seed fungi [63]. In the nested-PCR procedure, the sensitivity and specificity of detection of the target pathogen are significantly enhanced by performing a second round of PCRs using primers internal to the amplification product. As an example, nested PCR has been developed to detect the DNA levels of 10 pg for *Alternaria carthami* in safflower seeds [64]. However, this molecular assay is more laborious, more costly, and more prone to contamination than the conventional PCR [65]. Multiplex PCR allows for the detection of several seed pathogenic fungi simultaneously with high sensitivity. A triplex PCR was developed for the detection of fungal pathogens such as *Rhizoctonia solani*, *Fusarium oxysporum* forma specialis *ciceris,* and *Alternaria alternata* simultaneously isolated from pulse seeds, allowing for a quick and reliable detection of these pathogens [66]. The technique of MCH-PCR has the advantage that the magnetics beads are coated with single-stranded DNA probes and PCR amplification concentrates the DNA of interest while removing non-target DNA and other substrates that can inhibit the in vitro enzymatic of nucleic acids. This technique has been successfully used to detect *Botrytis* spp. in onion seeds [67]. The method of Bio-PCR consists of a pre-assay incubation step to increase the biomass of the fungal pathogen on the seeds, which is followed by DNA extraction and amplification by PCR. *Colletotrichum lupini* was diagnosed using the BIO-PCR method from lupin (*Lupinus* spp.) seeds [68]. Real-time PCR (qPCR) allows quantification of specific DNA targets by means of a fluorescent signal that is proportional to the amount of amplicon produced in each cycle. The fluorescent signal can be generated by an intercalating dye (SYBR green dye) or from the breakdown of a dye-labeled reporter probe (TaqMan labelled probes) during amplification. The TaqMan probe offers an extra level of specificity. However, qPCR needs a specialized instrument and the cost of the instrument and probe can be high [69]. A routine seed diagnosis for detecting *Didymella bryoniae* in cucurbit seeds using qPCR with a TaqMan probe has been published by the USDA-NSHS [70].

Despite their advantages, no PCR assay has yet been approved by the ISTA for fungi. Even when cost and expertise are not major limitations, technical impediments may lead to the occurrence of false negatives due to PCR inhibitors in seed extracts (tannins, phenolic compounds) or false positives originated by remnant DNA from non-viable propagules [59,71]. The inability to efficiently extract PCR-quality DNA from seeds has restricted the acceptance and application of PCR for the detection of seed pathogens [56].

The conventional PCR techniques also have other drawbacks such as the requirement of sophisticated instruments such as a thermocycler or a gel documentation system. To address these disadvantages, several isothermal amplification methods, such as loop-mediated isothermal amplification (LAMP), helicase dependent amplification (HDA), nucleic acid sequence-based amplification, etc., have been developed. LAMP assay has been widely used to detect fungal pathogens due to its high sensitivity and specificity compared to other conventional PCR methods [69], but the foldable microdevices are not available for any plant pathogens. Foldable microdevices can be effectively utilized at point-of-care centers for the rapid diagnosis of the plant pathogen without using any sophisticated instruments. Using the optimized LAMP assay conditions, a portable foldable microdevice platform was developed to detect *Magnaporthe oryzae* and *Sarocladium oryzae* in rice seeds [63]. This platform could serve as a prototype for developing on-field diagnostic kits to be used at the point-of-care centers for the rapid diagnosis of fungal pathogens in seeds.

High throughput sequencing (HTS), also termed next-generation sequencing (NGS), is an emerging technology in plant pathogen detection. HTS can generate massive amounts of DNA sequence data at very low cost. It is now technically and economically feasible to use HTS for the rapid detection of multiple target pathogens. HTS is an indirect seed health indirect method and should demonstrate the viability of the target organism and its pathogenicity [62]. This technology can also reveal the sequences of non-pathogenic commensal organisms that have no impact on seed health. The availability of more and more complete fungal genomes in public databases will contribute to the spread of HTS technologies for the accurate diagnosis of fungal plant pathogens and for discovering novel pathogens, especially those considered unculturable [72,73]. Therefore, these new technologies have many potential advantages in seed health testing and further work is required to investigate the use of HTS in seed health reference methods.

The technique of DNA barcoding uses a molecular diagnostic tool in which a small segment of DNA is used to identify the species. Barcode regions are universally found in target lineages and exhibit adequate DNA sequence variation to distinguish different species. The internal transcribed spacers (ITS) region of the nuclear ribosomal (rDNA) is used as a universal barcode region for fungi [74] due to the fact that it contains alternating areas of high conservation and high variability among fungal taxonomic levels [75]. These regions have also been commonly used for designing species-specific primers to detect and identify fungal pathogens. However, different studies have shown that although the ITS region is extremely useful as barcoding for many fungi, these sequences do not seem to perform well for species discrimination in some genera. The “Assembling the Fungal Tree of Life” project screened for prospective genetic markers as likely regions for fungal barcodes: ITS, cytochrome c oxidase subunit I (COI), the nuclear large ribosomal subunit, the nuclear small ribosomal subunit, the largest and second largest subunit of RNA polymerase II, elongation factor 1-α, the small subunit of the mitochondrial ribosomal operon, BenA (β-tubulin), actin, chitin synthase, calmodulin, and heat shock protein 90. Numerous studies reported that the resolution of such genetic markers provided good species resolution as compared to ITS [76]. It is possible to obtain advice on primer sequences from the Lutzoni lab link: http://lutzonilab.org/primer-sequences/ (accessed on 1 September 2022). The European and Mediterranean Plant Protection Organization (EPPO) has published a standard that describes the use of DNA barcoding protocols in support of the identification of several regulated fungi, comparing DNA barcode regions with those deposited in publicly available sequence databases (EPPOQ-bank, https://qbank.eppo.int/, accessed on 1 September 2022). Protocols describe the extraction of nucleic acids and the amplification of standardized markers [77].

### 3.3. Other Methods

Matrix-assisted laser desorption ionization time-of-flight mass-spectrometry (MALDI-TOF MS) is one of the sophisticated technologies that could be used for plant pathogen detection due to its rapid, simple and inexpensive sample pre-processing requisites and its lucid workflow. During mass analysis, the chemical analytes are ionized into charged molecules and the ratio of their mass to charge (*m*/*z*) is measured [78]. For the discrimination of *Tilletia controversa* from the *T. caries*/*T. laevis* complex, MALDI-TOF MS analysis of teliospores from bunt balls has proven to be a useful and fast tool during field inspections [79].

Recently developed tools based on multispectral vision systems can distinguish infected seeds from healthy seeds and it is also useful to determine the color, texture, and chemical composition of seed surfaces [56]. Multispectral imaging (MSI) combined with machine vision was used as a non-invasive alternative method to detect *Drechslera avenae* (*Helminthosporium avenae*) in black oat seeds (*Avena strigosa*) according to the reflectance, color, and texture features of the seed images [80].

Although the general principles of seed health testing of commercial seed lots are applicable to germplasm material in gene banks, the number of seeds stored is a common constraint for determining the sanitary status of the samples, as microorganism detection usually involves the destruction of relevant quantities of material. This poses a problem when the number of available seeds is small, which is frequent especially in large-seeded accessions. In these situations, Diekmann [81] points out that it may be desirable to recover viable seeds following testing (from blotter tests or washing tests) or to use standard germination tests for seed viability monitoring to detect certain pathogens. Some non-destructive assays have also been proposed to save genetic material [82,83].

Selection of the most appropriate test method should be based on the specificity of the assay together with the need to employ methodologies with sufficient sensitivity (Table 2). These factors, together with the availability of specialized laboratories or qualified personnel, will determine the final choice of analysis method, although the combination of different approaches seems to be the best option.

## 4. Fungi in Seed Germplasm Collections

### 4.1. Effect of Cold and Dry Storage on Fungi Longevity

The longevity of seed-borne fungi depends on diverse factors, including fungus type, its location in the seed, the severity of infection and the presence of antagonistic microbiota. However, storage conditions are the major factor affecting the survival of seed-borne fungi.

Several months or a few years of storage under normal room or warehouse conditions may substantially reduce the inoculum of many pathogens, and most of them were found to disappear after 10–15 years [3,44]. However, prolonging the period of commercial storage is generally not considered a practical method to free seeds from seed-borne fungi, due to the parallel decrease in seed viability. In a work by Russell [84], the longevity of *Helminthosporium sativus* (=*Bipolaris sorokiniana*, sexual morph *Cochliobolus sativus*) and *Alternaria tenuis* (=*A. alternata*) on wheat seed, stored under room conditions, was studied for 16 years. The viability of both fungi progressively decreased with time. However, *Alternaria* died out more rapidly and disappeared after 7 years, while *Helminthosporium* was found in a small percentage of seeds after 15 years, when the germination rate was below 30%. In an experiment conducted in Poland, fungi from *Fusarium* genera were not detected on barley grains after 3–5 years of uncontrolled storage, while *Bipolaris sorokiniana* and *Alternaria* were still present after this period [85].

In the case of gene banks, seeds are usually dried to a low moisture content and stored at low temperatures [86]. It has been well demonstrated that cold storage enhances the survival of seed-associated organisms, and, thus, gene banks may serve as reservoirs of seed-borne pathogens. Temperatures of −18 °C are recommended for the long-term conservation of germplasm [87]. Under similar sub-freezing conditions, Hewett [88] found that the viability of several seed-borne fungal pathogens was almost unaffected after 8–14 years of storage. *Ascochyta lentis* was also isolated from seed accessions stored at 4–6 °C for 33 years at the National Seed Storage Laboratory (NSSL) in the USA [89]. In a later study, Menzies et al. [90] found that mycelium of *Ustilago tritici* survived in infected wheat seeds after 32 years of storage at −15 °C. These authors point out that the conservation of infected seeds can be an advisable method to maintain collections of this fungus. Recently, data from the first 30 years of a 100-year seed storage experiment located in the Svalbard permafrost (−3.5 °C) have shown that all of the seed-borne pathogens have survived, and only a few of them have shown a reduction in the infection percentages [91].

In some cases, the increase in fungi survival at low storage temperatures has negatively affected the seed quality. Gilbert et al. [92] reported that *Fusarium*-infected wheat seeds had better germination and emergence rates after 24 weeks of storage at 10 °C and 20 °C than at −10 °C and 2.5 °C. Kaiser et al. [93] and Singh et al. [94] showed that the number of seeds infected by *Ascochyta fabae* and *Rhizoctonia bataticola* (=*Macrophomina phaseolina*) in lentils and chickpeas increased after several years of storage at sub-freezing temperatures. The reasons for these increases are not clear, because fungal growth cannot occur under these conditions. These results could be due, among other possibilities, to a shift in survival in other components of the mycoflora that formerly inhibited the growth of the fungus [93,95].

At the other extreme, the inverse relationship between temperature and fungal longevity has been widely used to control seed-borne pathogens through seed heat treatments [96,97,98,99].

To survive in seeds, many fungi are able to endure dehydration by producing xerotolerant propagules, such as drought-resistant conidia, chlamydospores, sclerotia or dormant mycelium [3], and desiccation tolerance has been established in the spores of a wide variety of fungi [100]. An inverse relationship between relative humidity and viability has been determined for the fungal spores of several entomopathogenic and phytopathogenic fungi [101]. In the study, a model of the effects of moisture content and temperature on conidia survival was developed. This model was analogous to that established for orthodox seeds [102], although the relative humidity range in which this model could be applied varied considerably among fungi. The relationship between relative humidity and fungal spore viability appears to be highly species-specific and more complex than in seeds. Thus, there are even examples of higher losses of conidia viability at intermediate relative humidity than at lower or higher values [103,104].

At the CRF-INIA gene bank, an experiment was conducted to test the effect of seed drying on seed-borne fungi on bean seeds. Seeds were dried at 13% and 5% relative humidity and 20 °C until a seed moisture equilibrium was reached. Desiccation treatments decreased the proportion of infected seeds for most of the fungi found (*Penicillium*, *Cladosporium*, *Alternaria*, *Ulocladium*, *Fusarium*, *Botrytis*, *Rhizoctonia*, among others). In contrast, the percentage of seeds in which *Aspergillus* and *Mucor* were detected tended to increase with seed drying [105].

Cold and low humidity storage environments also contribute to maintaining the viability of endophytic fungi of grass seeds [106,107]. Endophyte viability, however, generally decreases at a faster rate than seed viability [108].

In summary, the dry and cold storage conditions used in seed gene banks to increase seed longevity usually favor the survival of seed-borne mycoflora. Differences in fungi longevity under low temperatures and in sensitivity to desiccation might alter the initial composition of seed inoculums. This may have consequences on germinability that are difficult to predict after long storage periods, when seed susceptibility to diseases can also be higher due to the ageing process.

### 4.2. Risk of Pathogen Dissemination

According to the information compiled in the Second Report on the State of the World’s Plant Genetic Resources for Food and Agriculture [109], about 7.4 million germplasm accessions are conserved worldwide, the majority of which are maintained in seed collections. Gene banks play a central role in the movement of germplasm within and among countries, and hundreds of thousands of seed samples are supplied to plant breeders, researchers, or farmers each year. Data provided by the CGIAR [110] and the U.S. National Plant Germplasm System (NPGS) [111] indicate that these two gene bank networks alone distribute about 400,000 samples and more than 200,000 samples per year, respectively.

This extensive exchange of germplasm poses a risk of unintentionally introducing and disseminating plant pathogens by means of infected seeds [5]. Furthermore, the diversity of stored germplasm is often directly related to the diversity of seed-associated microorganisms. Thus, crop diversity centers are also expected to be areas of high variability of crop-specific pathogens, where exotic strains or races may occur [112]. In some seed-borne diseases, the introduction of compatible mating types from one region to another may lead to the development of the sexual state in new areas, and if this occurs the pool of virulence genes in the pathogen could be greatly increased [113].

Surveys carried out on seed lots received, conserved, or distributed by different national and international gene banks have revealed the presence of economically important pathogenic fungi. In Brazilian germplasm collections, 13 important pathogenic fungi were isolated from approximately 10,000 accessions of 24 crop species before long-term storage [114]. From 1982–1997 about 0.5% of the samples exported by the International Crops Research Institute for the Semi-Arid Tropics (ICRISAT) were detained due to severe fungal or bacterial infections [115]. The Seed Health Laboratory at the International Center for Agricultural Research in the Dry Areas (ICARDA) reported that 22.03% of incoming cereal seeds from 1995–2004 were infected with seed-borne pathogens, and *Tilletia caries* and *T. foetida* (=*T. laevis*), the common bunts of cereals, were the most frequently detected [116]. In India, during 2012–2014, thirty pathogenic fungi and one bacteria of quarantine significance were intercepted on the introduced germplasm and trial material of 63 crop species from 35 countries [117]. In 2018 and 2019, the Germplasm Health Unit (GHU) or the CGIAR centers tested and removed 7% of the 335,928 gene bank samples, including those for import, export, and regeneration, due to pest interception during phytosanitary processing [56]. 

Although seeds generally present a lower sanitary threat than vegetative material, germplasm recipients and providers should be aware of the phytosanitary hazards associated with seed exchange and take precautionary measures to minimize the risk of spreading pests and diseases. Bioversity International (formerly IPGRI and IBPGR) has published technical guidelines that contain useful information for safe germplasm transfer, two of which refer to the seeds of legumes and cereals [118,119].

To prevent the introduction of quarantine or regulated pests, most countries have legislation, regulations, or procedures which should be contemplated in the movement of germplasm. The IPPC (International Plant Protection Convention) is an international plant health agreement that coordinates regional and national phytosanitary measures and provides information related to import and export requirements, pest status and regulated pest lists provided by each member country (https://www.ippc.int, accessed on 1 September 2022).

The CGIAR gene banks, which conserve large amounts of germplasm from many geographical regions and send shipments to a broad range of countries, have a special concern for the safety of the transfer of materials. All CGIAR gene banks have GHUs that operate in coordination with the National Plant Protection Organization (NPPO) or the quarantine agency of the host country [5]. All newly introduced materials are subject to seed health checks before being included in the gene bank, and the health of outgoing seeds must also be controlled. The GHU of the International Rice Research Institute (IRRI) maintains a 12-hectare post-entry quarantine area for the initial planting of imported rice seeds, a genetic resource center nursery area for wild races, and a phytotron for transgenic materials. It makes crop health monitoring, treatment, and isolation of the imported germplasm more efficient and safer [120]. The guidelines followed by CGIAR gene banks for the safe transfer of germplasm are available in the CGIAR portal “Crop Genebank Knowledge Base” (http://cropgenebank.sgrp.cgiar.org, accessed on 1 September 2022).

According to Koo et al. [121] who provided information on gene bank costs in the CIMMYT (International Maize and Wheat Improvement Center), ICARDA, ICRISAT, IRRI and CIAT (International Center for Tropical Agriculture), the cost of seed health tests ranged from 4.2 to 18.8% of the annual operational costs of seed processing, conservation and distribution, with the lowest and highest values corresponding to IRRI and CIAT, respectively. In comparison, the operational costs of seed health testing were 1.4 (IRRI) to 3.6 (ICARDA) times higher than those corresponding to seed viability testing.

Fulfilling seed health requirements for international transfer can be a significant problem for many gene banks with limited resources and for many countries where there is a lack of technical and human resources for plant health inspection. Furthermore, national regulations often focus on commercial trade and large volumes of consignments and they are not adequate for the purposes of the international transfer of germplasm [5]. This situation has even led some national scientists to stop making requests as they know the plant material or seeds will be refused or will not reach them in a timely manner [122]. Ideally, quarantine services and germplasm scientists should cooperate to find a balance between protecting crops and promoting global agricultural research [4].

### 4.3. Effects of Fungi on Seed Longevity

The initial quality of samples, together with seed moisture level and storage temperature, are the main factors that influence the longevity of orthodox seed in gene banks [86]. Samples with high germination and vigor survive longer than low viability samples. Therefore, storing seeds of the highest initial quality is a priority of gene bank curators.

Seed infection by fungi in the field may be an important factor affecting seed quality. As mentioned in point (b) in Section 2.1, some pathogenic fungi that attack developing seeds while they are still on the plant (“field fungi”) can decrease seed viability. Extensive research has been carried out on the effects of these fungi on seed quality, and some recent references on this subject are provided in Table 3. In legumes, species of the complex *Phomopsis*/*Diaporthe* are among the most thoroughly studied seed-borne fungi due to their severe effects on yield and seed quality in soybean growing areas [123]. In grain cereals, *Bipolaris sorokiniana* and *Fusarium* spp. are major seed-borne pathogens which are widespread around the world and can reduce seed quality and cause seed rot and seedling blight [124,125]. In grasses, some fungal endophytes decrease seed longevity [126], and some species of *Alternaria* can negatively affect the seed quality of crucifers and other horticultural crops [127,128].

Some specific studies have been conducted under gene bank storage conditions to determine the effect of fungal pathogens on legume seed longevity. Groundnut seeds infected with *Macrophomina phaseolina* experienced a drastic decrease in germinability after five years of storage at 20, 4 and −18 °C, whereas healthy lots maintained their viability during the same period at all storage temperatures. Initial germination rates were also lower in infected lots than in healthy seeds (about 83% and 99%, respectively) [94]. Similar results have been obtained in lentil and chickpea seeds infected with *Ascochyta fabae* [93] and *A. rabiei* [135].

Other studies on seed germplasm point out the negative effects of some fungi on seed conservation, although they do not present correlations between fungal incidence and germination. Duan et al. [163] reported declines in germinability in Chinese gene bank wheat accessions infected with *Fusarium verticilliodes*, *Bipolaris nodulosa* and *Cladosporium herbarum*. In the germination tests of several species, Faiad et al. [114] noted that the main genera of pathogenic fungi isolated from abnormal seedlings were *Phoma*, *Pyricularia*, *Fusarium*, *Gerlachia*, *Diaporthe*, *Colletotrichum*, *Macrophomina,* and *Rhizoctonia*. A survey of seed-borne fungi on cruciferous seeds stored in a Japanese gene bank reported that seed lots were frequently infected by pathogens, especially by *Alternaria* spp., and *A. brassicicola* and *A. japonica* apparently inhibited seed germination and caused seed rot [164].

After harvest, storage fungi, mainly *Aspergillus* and *Penicillium*, may invade and deteriorate seed samples if drying is delayed and seeds are held under humid ambient conditions during the pre-storage period. Once the seeds have been desiccated to appropriate moisture contents for germplasm conservation, seed fungal growth is completely halted.

Many of the fungi that reduce seed quality are important producers of mycotoxins which are highly dangerous for human and animal health. *Fusarium* genus generates trichothecenes, zearalenone (*F. graminearum*, *F. culmorum*), and fumonisins (*F. verticilloides*, *F. proliferatum*, *F. subglutinans*). *Aspergillus flavus* is the main source of aflatoxin contamination, *Penicillium* spp. produce ochratoxin, and *Neotypodium* may originate a diverse array of alkaloids with antimammalian activities [11]. *Alternaria* species are also capable of producing several mycotoxins, including alternariol or tenuazonic acid [165]. Although comparatively fewer studies have been carried out on the effect of mycotoxins on plants than on human and animal health, these compounds can have negative effects on seed germination and seedling development [166,167,168,169].

In spite of the detrimental effect of some seed-borne fungi on seed quality, many other mycoflora commonly found in seeds do not have a negative influence on seed viability. For example, *Alternaria* is almost always present as mycelium beneath the pericarp of wheat but normally cause no seed quality problems [7].

**Figure 3:**
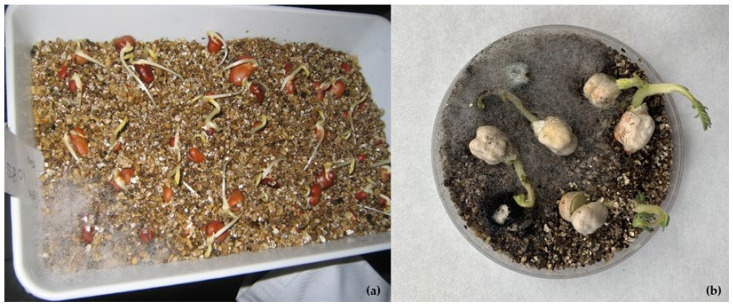
Germination test in vermiculite substrate. Fungi growth remains localized around the dead tissues: (**a**) bean seeds with growth of *Rhizopus*; (**b**) chickpea seeds with growth of *Rhizopus*, *Aspergillus,* and *Penicillium*.

Another aspect that should be considered in this section is the effect of fungal contamination on germination tests carried out in gene banks to periodically monitor seed viability. Germination tests are performed in temperate and humid conditions that favor the rapid growth of saprophytic fungi such as *Rhizopus*, *Penicillium,* or *Aspergillus*. The initial inoculum may be in the seeds themselves, in the substrates, or in the air. Saprophytic fungi can readily colonize dead or weakened seeds and then spread to healthy seeds. Thus, seeds that are initially viable may die or produce abnormal seedlings due to secondary infections, leading to an underestimation of the viability of the seed lot, which may result in unnecessary field regenerations of the samples. Especially in large-seeded legumes, the use of sand or similar substrates is a very effective method for reducing fungal spreading in germination tests (Figure 3).

## 5. Procedures to Reduce Harmful Effects of Fungi in Gene Banks

As described in previous sections, pathogenic fungi associated with gene bank seed accessions may pose a risk of disseminating diseases and reducing seed longevity. Furthermore, characterization and evaluation tasks may be affected by the use of infected material. Hence, seeds stored in gene banks should be as free of pathogens as possible. To accomplish this, precautionary measures should be taken at different stages of ex situ management: material acquisition, multiplication/regeneration, and seed processing. Thorough and comprehensive procedures aimed at guaranteeing seed health are provided in the protocols established at CGIAR gene banks [170,171,172,173] or at the National Bureau of Plant Genetic Resources, India [174].

- Material acquisition:

Materials collected or acquired outside the gene bank country should meet national phytosanitary regulations to ensure that the incoming seeds are free from pathogens of quarantine significance. Other pathogens may already be established in the country, but even in this case, the introduction of new races may be a risk factor. Special care must be taken when the seeds have been collected in the wild or from farmers in centers of crop diversity.

Many times, gene banks do not have the resources necessary to assess the health status of the seeds received. Then, it is important, when possible, to obtain samples from institutions that can provide reliable phytosanitary certificates. In any case, the first propagation should be carried out in an isolated area, and the plants must be regularly checked for signs of disease. Appropriate seed treatments (fungicides, heat) can be applied before sowing to eradicate potential seed-borne pathogens. Finally, when risky infected materials cannot be cleaned, they must be destroyed.

- Regeneration/Multiplication:

Regeneration/multiplication is the gene bank operation that has the greatest influence on seed health quality. Appropriate agronomic practices and, especially, adequate crop rotation should be established to reduce the incidence of disease in field plots. The technical experts responsible for regeneration activities should be familiarized with the symptoms of the most important diseases that can be seed-transmitted or affect seed quality. The section “Management strategies/Safe Transfer of Germplasm/Guidelines” of the CGIAR-Crop Gene bank Knowledge Base provides disease descriptions for barley, chickpea, common bean, cowpea, faba bean, forages, groundnut, lentil, maize, millet, pigeon pea, rice, sorghum, and wheat (http://cropgenebank.sgrp.cgiar.org, accessed on 1 September 2022). If necessary, phytosanitary treatments should be applied, preferably environmentally-friendly treatments. The removal of infected plants is a general agronomic practice to limit the spread of disease, but, in the case of heterogeneous accessions, the elimination of large numbers of individuals can reduce within-sample diversity. Klein and Wyatt [175] reported important diversity declines in samples of the CIAT bean collection after three growth cycles during which individual plants infected with bean common mosaic virus were eliminated.

When the environmental conditions of the gene bank field plots favor the development of seed-borne diseases, it is better to carry out regeneration at other more appropriate sites. Likewise, it is always preferable to discard heavily infected seed lots from an unfavorable harvesting season and perform a new regeneration cycle.

- Seed processing:

Once seed samples have been collected or harvested, environmental conditions during transport and seed processing may give rise to further fungal infections or contaminations. One such example occurred in Spain with maize samples that were regenerated in a humid coastal area and sent to the CRF-INIA gene bank in vacuum-sealed plastic bags. During transport, some of the bags lost their vacuum seal, causing high humidity levels inside them which led to fungal growth on the seeds and drastic losses of germinability.

The pre-storage period before seed drying should be as short as possible, especially in humid climates. Even in dry climates, seed lots may suffer localized high humidity conditions that can promote seed fungal spoilage, e.g., when seeds are in prolonged contact with fresh plant debris. As a general rule, orthodox seeds with high moisture contents should never be maintained in plastic bags or other watertight containers. Open trays, paper, or cloth bags can be used instead.

Threshing and cleaning equipment, trays, tables, and all seed handling materials should be properly cleaned to avoid contamination with microorganisms that may be present in soil or plant residue. Bleach is an economic and effective disinfection agent that is widely used. Periodic checking of the sanitary status of the seed processing sites is strongly advised. Air-borne spores can be sampled by placing open plates with culture media in laboratories and processing chambers. When pathogens are detected, room disinfection can be easily carried out by means of commercial aerosols for air treatment.

Special cleanliness must be maintained in the activities related to seed viability monitoring, as the environment of the germination tests is suitable for fungal development. Seeds should be properly spaced, and decaying seeds should be quickly removed to prevent the spread of fungi to other seeds. Sand may be a better substrate than paper towels in the evaluation of diseased samples.

Whenever possible, seed health tests should be conducted on representative samples before storage. A practical approach to save materials is to evaluate preliminary seed health during routine germination tests. Then, specific health tests can be carried out only on samples suspected of having seed-borne pathogens. In general, heavily infected seed lots are not appropriate for long-term storage. For example, at the ICRISAT, seed samples are considered unsuitable for conservation as a base collection, if more than 5% of the seeds are infected by one or more of the following fungi: *Alternaria*, *Aspergillus*, *Cladosporium*, *Curvularia*, *Fusarium*, *Macrophomina*, *Penicillium*, *Phoma* and *Rhizopus* [171]. Samples with aggressive pathogens that can be seed-transmitted should be excluded from seed distribution activities.

Chemical, physical or biological eradication of seed-borne fungi may improve germination levels [176,177], although seed quality may not be entirely restored due to the damage previously caused by the infection. Consequently, it might be expected that seed treatment prior to storage could be beneficial for seed longevity. However, the use of chemical treatments during seed storage in gene banks is not recommended, as they can have long-term toxic effects on the genetic material [178].

Among physical treatments, heat application, or thermotherapy, has been widely used to decrease seed-borne infections [96,97,98,99] although this procedure almost inevitably has some negative effects on seed vigor and germination. In this sense, temperatures and exposure times that are effective for pathogen elimination without affecting seed germination will be largely dependent on the pathogen, the plant species, the cultivar, the seed moisture content, and the age of the seed lot. Hence, the use of seed thermotherapy prior to gene bank storage does not seem advisable as a routine method of seed sanitation, although it might be useful in some cases.

The situation is similar for essential oils, whose antifungal activity has been documented in numerous studies [179], but their application is often associated with adverse effects on the seed germination process [180].

The use of thermotherapy or plant products for disinfecting seeds is an emerging area of research of special interest for organic farming systems [181]. However, to evaluate their usefulness in long-term seed preservation, additional research is needed to investigate the effect of these treatments on seed longevity. It should be borne in mind that other treatments, such as seed hydration–dehydration (seed priming), aimed at improving germination performance, often reduce seed lifespan [182].

## 6. Conclusions and Future Perspectives

The uncontrolled movement of infected seeds can lead to the rapid spread of pests and diseases. Seed health testing and phytosanitary clearances are mandatory and regulated processes under the conventions of the IPCC and the respective policies of the NPPOs. Fungi may survive in seeds for decades under the dry and cold conditions of gene banks. Seed-importing countries need assurance that the plant germplasm that enters their borders is free from any pathogens or pests of quarantine importance. Therefore, intensive efforts are needed to develop more seed health test methods for increasing specificity, sensitivity, speed, simplicity, cost effectiveness, and reliability in the diagnosis of fungal pathogens in seeds. Molecular methods based on PCR assay and HTS could contribute to this goal. Pathogenic fungi associated with gene bank seed accessions can reduce seed quality and longevity, so stored seeds should be as free of pathogens as possible. Preventive measures should be taken at different stages of ex situ management. These measures have to be adapted depending on the seed and type of pathogen present.

Further studies on the longevity and pathogenicity of seed-borne fungi after long-term storage would be required to obtain a better understanding of the possible effects of fungal infections on the viability of seeds stored for decades in gene banks. Moreover, the benefits or drawbacks of seed treatment before long-term storage remain largely unexplored.

In spite of the detrimental effects of some seed-borne fungi on seed quality, many other endophytic mycoflora do not have a negative influence on seeds but may have a role in controlling pathogens, among other aspects, therefore they should be studied in depth.

## Figures and Tables

**Table 1:** Minimum relative humidity and seed moisture content for the growth of common storage fungi (from Meronuk [32]).

Relative Humidity (%)	Fungi	Seed Equilibrium Moisture Content ^1^ (%)		
		Starchy Cereals	Soybeans	Sunflower, Safflower, Peanut
65–70	*Aspergillus halophilicus*	13.0–14.0	12.0–13.0	5.0–6.0
70–75	*A. restrictus*, *A. glaucus*, *Wallemia sebi*	14.0–15.0	13.0–14.0	6.0–7.0
75–80	*A. candidus*, *A. ochraceus*, plus the above	14.5–16.0	14.0–15.0	7.0–8.0
80–85	*A. flavus*, *Penicillium*, plus the above	16.0–18.0	15.0–17.0	8.0–10.0
85–90	*Penicillium*, plus the above	18.0–20.0	17.0–19.0	10.0–12.0

^1^ Figures are approximations, in practice, variations up to ±1% can be expected.

**Table 2:** Mean features of seed health methods including time required form completion, sensitivity, ease of application, specificity, and ease of implementation. Modified from Kumar et al. [56].

Type of Assay	Time Required	Sensitivity	Ease of Application	Specificity	Ease of Implementation
Visual examination	<10 min	Low	Simple and inexpensive	Low	Mycological skills required
Staining method	<10 min	Low–moderate	Simple and inexpensive	Low–moderate	Mycological skills required
Embryo extraction	<10 min	Low–moderate	Inexpensive	Low–moderate	Mycological skills required
Seed washing test	<30 min	Low	Simple and inexpensive	Low	Mycological skills required
Agar plating	5–7 days	Moderate	Simple and inexpensive	Low–moderate	Mycological skills required
Blotter test	1 week	Moderate	Simple and inexpensive	Moderate	Mycological skills required
Seedling symptom test	2–3 weeks	Low	Simple and inexpensive	Low	Mycological skills required
Pathogenicity test	2–3 weeks	Moderate	Inexpensive	Low	Mycological skills required
Serology-based assay (ELISA)	2–4 h	Moderate-high	Simple, moderately expensive and robust	Moderate-high	Ease of interpretation
PCR conventional	5–6 h	High	Complicated ease of interpretation and expensive	Very high	Molecular biology skills required and ease of interpretation
Nested PCR	5–6 h	Very high	Complicated and expensive	High	Molecular biology skills required and ease of interpretation
Multiplex PCR	5–6 h	High	Complicated ease of interpretation and expensive	Very high	Molecular biology skills required and ease of interpretation
Real time PCR	40–60 min	Very high	Complicated and expensive	Very high	Molecular biology skills required
MCR-PCR	2–5 h	Very high	Complicated and expensive	Very high	Molecular biology skills required
Bio-PCR	5–7 days	Very high	Complicated and expensive	Very high	Molecular biology skills required and ease of interpretation
LAMP assay	<4 h	High	Expensive	Very high	Molecular biology skills required and ease of interpretation. Portable devices.
HTS	4–7 days	High	Complicated and very expensive	High	Molecular biology and bioinformatic skills required
DNA barcoding	4–7 days	High	Complicated and expensive	Very high	Molecular biology and bioinformatic skills required
MALDI-TOF MS	5–6 h	High-moderate	Simple, moderately expensive	High	Ease of interpretation
MSI	<2 h	Moderate	Simple	Low	Technological skills required

ELISA: enzyme-linked immunosorbent assay; PCR: polymerase chain reaction; MCR-PCR: magnetic capture hybridization-polymerase chain reaction; Bio-PCR: biological and enzymatic polymerase chain reaction; LAMP: loop-mediated isothermal amplification; HTS: high throughput sequencing; MALDI-TOF MS: matrix-assisted laser desorption ionization time-of-flight mass-spectrometry; MSI: multispectral imaging.

**Table 3:** Field fungi that affect seed viability and references.

Host	Fungus	Country	References
*Brassica rapa*	*Alternaria brassicae*, *A. raphani*	Canada	Rude et al. [129]
Barley	*Fusarium graminearum*	Canada	Tekauz et al. [130]
	*Fusarium* spp. and others	Slovakia	Hudec [131]
Carrot	*Alternaria* spp.	Serbia	Bulajić et al. [132]
	*Alternaria alternata*	China	Zhang et al. [133]
Cauliflower	*Alternaria brassicicola*, *A. brassicae*	The Netherlands	Köhl et al. [134]
Chickpea	*Ascochyta rabiei*	Pakistan	Ahmad et al. [135]
Groundnut	*Aspergillus flavus*, *A. niger* and others	India	Kar et al. [136]
Legumes	*Macrophomina phaseolina*	Review	Pandey et al. [137]
*Lolium multiflorum*	*Neotyphodium occultans*	Argentina	Gundel et al. [138]
*Lolium perenne*	*Fusarium* spp., *Bipolaris sorokiniana* and others	China	Zhang et al. [139]
Maize	*Fusarium verticilloides*	USA	Yates et al. [140]
	*Fusarium verticilloides*	Nigeria	Anjorin et al. [141]
	* Aspergillus flavus *	USA	Dolezal et al. [142]
	* Stenocarpella maydis *	Brazil	Siquiera et al. [143]
Oat	*Fusarium graminearum*	Norway	Tekle et al. [144]
Rapeseed (*Brassica napus*)	*Alternaria* spp.	Pakistan	Soomro et al. [145]
Rice	* Sarocladium oryzae *	India	Gopalakrishnan et al. [146]
	* Bipolaris oryzae *	Brazil	Schwanck et al. [147]
Sorghum and foxtail millet (*Setaria italica*)	*Alternaria*, *Curvularia*, *Fusarium* and others	South Korea	Yago et al. [148]
Soybean	*Diaporthe species complex*	USA	Petrović et al. [149]
	*Diaporthe species complex*	Paraguay	Mengistu et al. [150]
	*Diaporthe species complex*	Korea	Sun et al. [151]
	*Colletotrichum truncatum*	Brasil	Da Silva et al. [152]
	*Fusarium verticilloides*	USA	Pedrozo and Little [153]
	*Fusarium* spp.	Italy	Ivic [154]
	*Cercospora kukuchii*	USA	Turner et al. [155]
Wheat	Black point (*Alternaria triticina, Bipolaris sorokiniana, Fusarium graminearum*)	India	Sharma et al. [156]
	*Fusarium graminearum*	Iran	Hassani et al. [157]
	Black point (*Alternaria alternata*, *Bipolaris sorokiniana*, *Fusarium graminearum*)	Egypt	El-Gremi et al. [158]
	*Fusarium culmorum*, *Microdochium nivale*	UK	Haigh et al. [159]
	*Fusarium* spp., *Microdochium nivale*	Slovakia	Hudec and Muchová [160]
	*Bipolaris*, *Fusarium*, *Thielavia*, and others	Iran	Sharafi et al. [161]
	*Alternaria* spp. Complex	Argentina	Perelló and Larran [162]

## Data Availability

Not applicable.
